# Editorial: Understanding the link between sleep and mental health

**DOI:** 10.3389/frsle.2024.1498365

**Published:** 2024-11-05

**Authors:** Yuen Mi Cheon

**Affiliations:** Department of Family Environment and Welfare, Chonnam National University, Gwangju, Republic of Korea

**Keywords:** sleep, mental health, psychological wellbeing, emotion, cognition, sleep quality, sleep disturbance

Over the years, researchers have examined and confirmed that sleep is an essential part of one's physical and mental health. Both sleep quantity and quality have been found to be a meaningful predictor of various aspects of mental health (Bacaro et al., 2024; Freeman et al., 2017; Scott et al., 2021; Short et al., 2019). While these studies have successfully started to uncover the links between sleep and mental health, the intricate details in various aspects of sleep and mental health across various contexts have yet to be discovered. The articles included in this Research Topic, “*Understanding the link between sleep and mental health*” are the latest research that address this gap and contributes to elaborating the current state of our understanding of sleep and mental health. This editorial summarizes the main findings of these studies, highlights the main contributions and provide suggestions for future research.

Kabunga et al. examined burnout and coping mechanisms among healthcare professionals in central Uganda. They found a high prevalence of burnout, active coping, positive reframing, and denial, dysfunctional coping to be correlated with burnout, while emotion-focused coping was not. Counterintuitively, a little more than half of the participants (56.9%) in this study reported having adequate sleep. Coelho et al. sought to validate a French version of Sleep Beliefs Scale (SBS). The French version of SBS in their study were appropriately translated and could be validated. Theses scores were associated with depression but not anxiety. Pal et al. examined the association between childhood abuse and obstructive sleep apnea (OSA) among the women in the United States. They found that early childhood sexual abuse was 2.4 times more commonly reported among women with OSA. Also, BMI mediated the link between early childhood physical abuse and OSA. In Tari et al., the link between sleep and cognition was examined in a study called PREVENT, which included participants in the United Kingdom and Ireland. They found sleep quality and sleep quantity to be related to participants' depressive symptoms; participant apathy to sleepiness and sleep-disordered breathing. The link between sleep and cognition was mediated by depressive symptoms. Lajunen et al. conducted a study that included 52 countries to examine the link between sleep and happiness. They found a relatively strong correlation between national average of sleep duration and national average of subjective wellbeing. Governance quality, schooling and obesity were strongly related to sleep. Sleep was also related to power distance, individualism, masculinity. Lastly, Saeda et al. conducted a study to examine the effects of pleasant sound on overnight sleep condition in Japan. They found sleep onset latency to be shorter with pleasant sound than white noise among the sleep maintenance difficulty group, while no association was found for the entire sample. Also, the number of spindles and the spindle density were associated with the pleasant sound.

The studies in this Research Topic have covered a variety of population around the world and revealed various types of links between sleep and mental health. The countries included in these studies were Uganda (Kabunga et al.), France (Coelho et al.), Japan (Saeda et al.), UK and Ireland (Tari et al.), US (Pal et al.), as well as 52 countries (Lajunen et al.). Although each of these studies focus on unique aspects of the sleep-mental health link, their results imply that this link is a globally relevant phenomenon that requires further attention within a variety of national contexts—possibly comparing the similarities and differences across nations and cultures.

The unique aspects of the sleep-mental health links examined in these studies also contribute to the existing literature by deepening and widening our perspective providing creative ideas for multitude of follow-up studies. Kabunga et al. suggests a potential role of coping strategies in the link between adequate sleep and burnout among healthcare professionals in Uganda. Unfortunately, this study did not specifically examine the direct association between adequate sleep and burnout as well as the moderating role of coping. Examining these associations will provide helpful information for prevention and intervention. Coelho et al.'s study has opened the door for numerous follow-up studies on sleep beliefs in the French context. Further studies could also validate the use of this scale among other national and cultural populations. Pal et al. extends the existing knowledge of obstructive sleep apnea by examining the role of childhood abuse in women. Further studies could examine the longitudinal role of childhood experiences and environments in sleep across the development. Tari et al. provides evidence for the role of sleep in emotional as well as cognitive aspects of mental health, while Lajunen et al. provides possible correlates of sleep and mental health at socio-economic, population and cultural levels, suggesting a more sophisticated models with multi-level analyses. Lastly, Saeda et al. suggests a new area of sleep intervention—pleasant sound. Further studies could examine various types of specific sensory interventions to help enhance the quality and quantity of sleep.

Overall, the studies in this Research Topic have made contributions especially by providing a global perspective and suggesting unique aspects of sleep-mental health links. It is hoped that future studies would utilize these findings to extend our knowledge of the link between sleep and mental health even further.
